# RBC Adherence of Immune Complexes Containing Botulinum Toxin Improves Neutralization and Macrophage Uptake

**DOI:** 10.3390/toxins9050173

**Published:** 2017-05-19

**Authors:** Fetweh H. Al-Saleem, Rashmi Sharma, Rama Devudu Puligedda, Md. Elias, Chandana Devi Kattala, Paul M. Simon, Lance L. Simpson, Scott K. Dessain

**Affiliations:** 1 Lankenau Institute for Medical Research, 100 E. Lancaster Ave, Wynnewood, PA 19096, USA; walsaleem@yahoo.com (F.H.A.-S.); rashmikaushik1@gmail.com (R.S.); ramdev.puligedda@gmail.com (R.D.P.); chandu.devi@gmail.com (C.D.K.); simonpm@aol.com (P.M.S.); 2 Inventox, PMB #172, 7 Avenida Vista Grande B-7, Santa Fe, NM 87508, USA; mdelias500@yahoo.com (M.E.); btkidone@gmail.com (L.L.S.)

**Keywords:** biodistribution, botulinum toxin, clearance, immune complex, macrophage activation, mechanism, monoclonal antibody, neutralization, pharmacokinetic, red blood cell

## Abstract

In the paralytic disease botulism, the botulinum neurotoxin (BoNT) passes through the bloodstream to reach and inactivate neuromuscular junctions. Monoclonal antibodies (mAbs) may be useful BoNT countermeasures, as mAb combinations can rapidly clear BoNT from the blood circulation. We have previously shown that the BoNT-neutralizing potency of mAbs can be improved through red blood cell (RBC) immunoadherence. For example, a fusion protein (FP) that adheres biotinylated mAbs to the RBC surface enabled a pair of mAbs to neutralize 5000 LD50 BoNT/A in the mouse protection assay. Here, we added two mAbs to that combination, creating a 4-mAb:FP complex that neutralized 40,000 LD50 BoNT/A in vivo, and analyzed functional correlates of neutralization. The FP enhanced potency of BoNT/A immune complexes, providing the greatest magnitude of benefit to the 4-mAb combination. RBC binding of a BoNT/A complexed with 4-mAb:FP exhibited a bi-phasic clearance process in vivo. Most of the complexes were cleared within five minutes; the rest were cleared gradually over many hours. Peritoneal macrophages showed better uptake of the 4-mAb complex than the 3-mAb complex, and this was not affected by the presence of the FP. However, the addition of RBCs to the 4-mAb:FP BoNT/A doubled macrophage uptake of the complexes. Lastly, the 4-mAb:FP BoNT/A complex synergistically induced M2 macrophage polarization, as indicated by IL-10 expression, whether or not RBCs were present. RBC-targeted immunoadherence through the FP is a potent enhancer of mAb-mediated BoNT/A neutralization in vivo, and can have positive effects on BoNT/A sequestration, immune complex uptake, and macrophage activation.

## 1. Introduction

Despite widespread progress in antimicrobial drug development, emerging infectious diseases, drug-resistant pathogens, and toxin-producing pathogens remain a considerable global concern. Monoclonal antibodies (mAbs) have proven to be safe and effective as therapeutics for treating infectious diseases and toxins, yet their broad potential has been largely unrealized. This is partly because single mAbs have limited utility against pathogens that are antigenically diverse, have redundant mechanisms of virulence, or require oligomeric immune complex formation for clearance. Combinations of mAbs that have complementary binding and neutralizing activities may be able to circumvent these problems. We have been studying immune complex mediated clearance in vivo using neutralization of botulinum neurotoxin (BoNT) as a model system.

BoNT is the most potent biological toxin known, and is ranked by CDC as a Category A Select Bioterror agent [1]. In the disease of botulism, BoNTs enter the body by crossing respiratory or intestinal mucosal epithelia, after which they transit through blood circulation to reach cholinergic nerve endings in neuromuscular junctions (NMJ) [2]. Upon entering the pre-synaptic neurons, they block the release of acetylcholine and induce neuromuscular paralysis. Immune complexes formed between BoNT antisera or combinations of monoclonal antibodies can inhibit BoNT toxicity by inducing rapid clearance of BoNT from the blood circulation [3]. Clearance results from BoNT immune complex uptake by macrophages in the liver, and functions in parallel to inhibition of BoNT activity at the NMJ. Effective IgG-mediated clearance of BoNT requires a minimum of three or more IgG Fc domains and can be effective against tens of thousands of lethal doses (LD50) in mice [4,5].

We have been studying approaches to enhance the in vivo clearance of antibody:BoNT immune complexes, with a goal of increasing the in vivo neutralization activity of mAbs that bind important epitopes, but lack significant BoNT inhibitory activity. Immune complex clearance from the blood circulation likely utilizes the complement system, in which immune complexes are opsonized by C3b and bind to Complement Receptor 1 (CR1) on the surface of RBCs, from which they are delivered to macrophages in the liver and spleen [6]. RBC adherence in itself may provide a protective function in vivo by preventing BoNT from reaching the NMJ. We have tested two different strategies for inducing sequestration of BoNT immune complexes on RBCs. We converted a pair of BoNT-specific mAbs into “Heteropolymers” (HP); hybrid molecules consisting of a mAb specific for CR1, chemically cross-linked to another mAb specific for the target pathogen [7,8]. We found that the pair of BoNT-specific HPs significantly enhanced neutralization, in part through sequestration of BoNT on the surface of RBCs. Macrophage uptake of HP-bound BoNT was significantly better with four Fc domains than with three. We also tested a novel fusion protein (FP), which consists of an scFv specific for the RBC surface protein, glycophorin A (GPA), fused to streptavidin. The FP induced RBC adherence of BoNT bound to pairs of biotinylated mAbs, and substantially increased their in vivo neutralizing activity [9].

Here we extend our study to explore factors that contribute to clearance of RBC adherent immune complexes from the blood circulation. Using the FP as a tool, we reformatted the configuration of the immune complex using two, three and four non-overlapping mAbs at concentrations that enabled the effects of RBC adherence to be measured. We then studied the contribution of the individual components to BoNT neutralization in vivo, the time course of BoNT clearance in vivo, and the process of macrophage uptake in vitro. Our findings examine general principles of toxin handling that will inform the design of neutralizing mAb cocktails against BoNT and other diverse toxins.

## 2. Results

### 2.1. Assembly of an Immune Complex with RBC Adherence and High-Potency BoNT/A Neutralization

**Figure 1:**
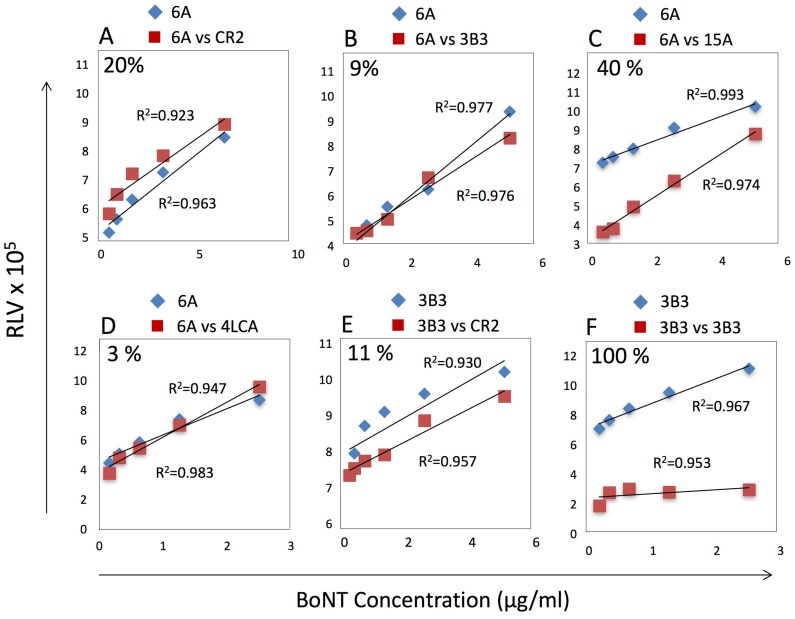
Competitive binding assays. Binding of biotinylated 6A or 3B3 mAbs to serial dilutions of RI-BoNT/A was assessed in the absence of potential competitor mAbs. Assays tested inhibition of biotinylated 6A binding in the presence of un-modified mAbs: CR2 (**A**), 3B3 (**B**), 15A (**C**) and 4LCA (**D**), or the inhibition of biotinylated 3B3 binding in the presence of CR2 (**E**) or 3B3 (**F**). Percent reduction in binding is shown in the top left of each panel and was calculated using the trapezoidal method.

In our previous study, we observed neutralization of 2000 LD50 with biotinylated 6A and 4LCA mAbs bound to the FP. We sought additional mAbs that would enable us to study the impact of higher-order complexes on RBC-dependent BoNT/A neutralization and clearance in vivo. Our goal was to create a multimeric FP-containing complex with neutralization potency comparable to the best results reported by others, i.e., 40,000 LD50 BoNT/A [4,5]. We tested three BoNT/A heavy chain (HC)-specific mAbs for their ability to bind recombinant inactive BoNT/A (RI-BoNT/A) in the presence of the 6A and 4LCA mAbs. 3B3 is a human mAb that binds BoNT types A, B and E, but is not neutralizing; CR2 is a humanized mAb that neutralizes type BoNT/A in vivo with high potency; and 15A is a human mAb with modest BoNT/A neutralization activity [8,9,10]. We measured binding of biotinylated 6A to a BoNT/A dilution series in the presence or absence of the potential competitor mAbs (Figure 1), and calculated the percentage of binding inhibition using the linear trapezoidal approximation method [11]. CR2 and 3B3 did not prevent 6A from binding to BoNT/A (Figure 1A,B), whereas 15A did compete with 6A (Figure 1C). As expected, the 4LCA, which binds the BoNT/A light chain (LC), did not compete with 6A (Figure 1D). Furthermore, CR2 did not compete with 3B3 binding (Figure 1E), whereas 3B3 did block its own binding (Figure 1F). Reciprocal competition studies gave similar results (data not shown). We therefore selected the following mAb combinations for further testing: 2-mAb (4LCA + 6A), 3-mAb (4LCA + 6A + 3B3), and 4-mAb (4LCA + 6A + 3B3 + CR2).

We tested these mAb combinations for BoNT/A neutralization in mice, with and without the FP [9,12]. Complexes were assembled using a step-wise protocol that first bound 6A and 4LCA to BoNT/A, then any additional mAbs, and then the FP. For the 3-mAb and 4-mAb complexes, we biotinylated 3B3, and for the 2-mAb complex, we biotinylated 6A. We then empirically determined by titration the lowest amounts of these components that would afford the 4-mAb:FP complex with 40,000 LD50 in vivo neutralization potency: 20 μg 6A, 20 μg 4LCA, 20 μg 3B3 biotin, 2 μg CR2, and 30 μg FP (data not shown). The lower dose of CR2 was enabled because of its extremely high potency (200 LD50 BoNT/A neutralization by 50 μg in vivo; Dr. Jianlong Lou and Dr. James Marks, personal communication).

Having established these quantities for each reagent, we tested the combinations against increasing doses of BoNT/A in vivo (Table 1). Against 5000 LD50, 2-mAb and 3-mAb both failed to provide 100% neutralization, whereas 4-mAb fully neutralized 5000 LD50 and partially neutralized 7500 LD50 (30%). The addition of the FP increased the potency of all of these combinations. The 2-mAb:FP and 3-mAb:FP complexes neutralized 5000 LD50, and the 3-mAb:FP gave 30% activity against 7500 LD50. 4-mAb:FP achieved 100% neutralization of 40,000 LD50. Notably, the FP had a greater absolute effect on the 4-mAb complex than on the 3-mAb. For example, the 4-mAb:FP provided full survival at a dose of 35,000 LD50 BoNT greater than the maximum dose that enabled full survival with the 4-mAb alone (e.g., 40,000 LD50 vs. 5000 LD50). Thus, the in vivo neutralization potency correlated with the number of mAbs in the complex, and this benefit was further enhanced by the FP.

### 2.2. Adherence of the 3-mAb:FP and 4-mAb:FP Complexes to RBCs In Vitro and In Vivo

**Figure 2:**
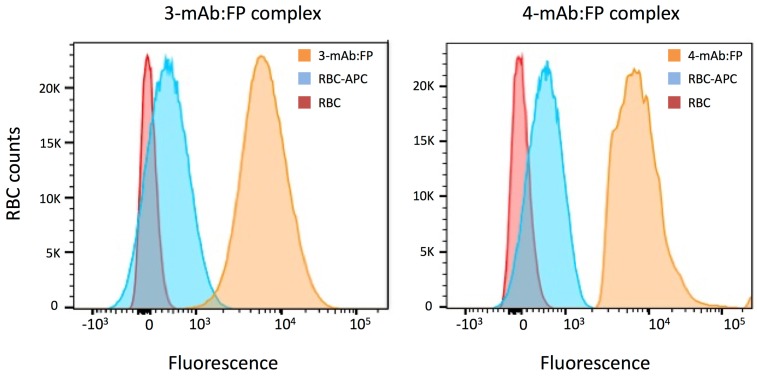
Binding 3-mAb:FP and 4-mAb:FP immune complexes to RBCs in vitro. Washed Swiss Webster RBCs were incubated with RI-BoNT/A bound to 3-mAb:FP (**left**) or 4-mAb:FP (**right**). Complex binding to RBCs was detected using an APC-conjugated anti-human IgG and flow cytometry. Tracings include RBCs only (red), RBCs with the APC anti-human IgG (blue), and RBCs with the BoNT/A complexes and the APC anti-human IgG (orange).

**Figure 3:**
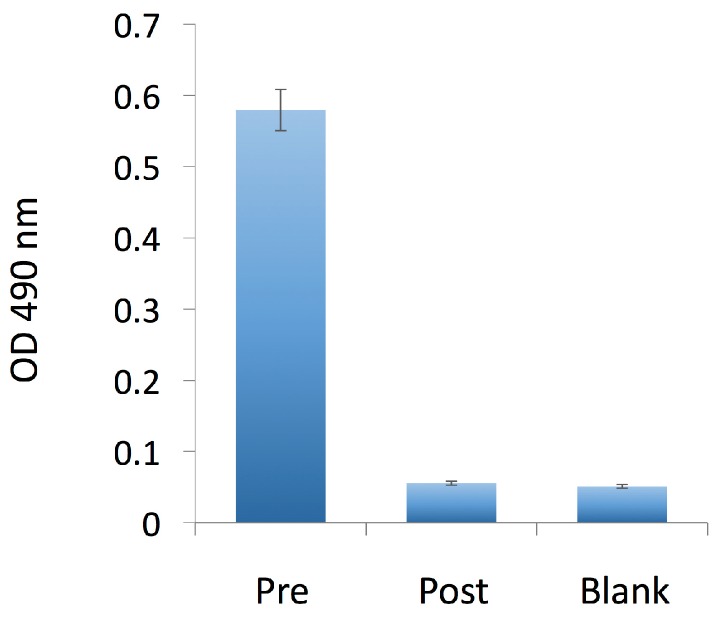
Qualitative assessment of BoNT/A in solution before and after incubation with murine RBCs. RI-BoNT/A contained in the 3-mAbs:FP complex was detected by ELISA in a solution before (Pre) and after (Post) its incubation with the RBCs, as shown in Figure 2, and compared to a buffer-only sample (Blank). Error bars indicate the S.E.M.

We next assessed the interaction of the 3-mAb:FP and 4-mAb complexes with RBCs in vitro and in vivo. We assembled the complexes in vitro with 200 ng RI-BoNT/A (40,000 LD50 equivalent) then incubated them for 1 h at room temperature with murine RBCs. We washed the RBCs and measured bound complex by flow cytometry with an APC anti-human IgG secondary mAb (Figure 2). Both the 3-mAb:FP and 4-mAb:FP complexes uniformly labeled the RBCs with approximately the same fluorescence intensity. We qualitatively compared the amount of RI-BoNT/A in the 3-mAb:FP + RBC binding reaction before and after RBC binding, using an ELISA with rabbit anti-BoNT/A HC50 antiserum [13]. Following RBC binding, the amount of RI-BoNT/A in solution was reduced to below the detection limit of the assay (Figure 3).

**Figure 4:**
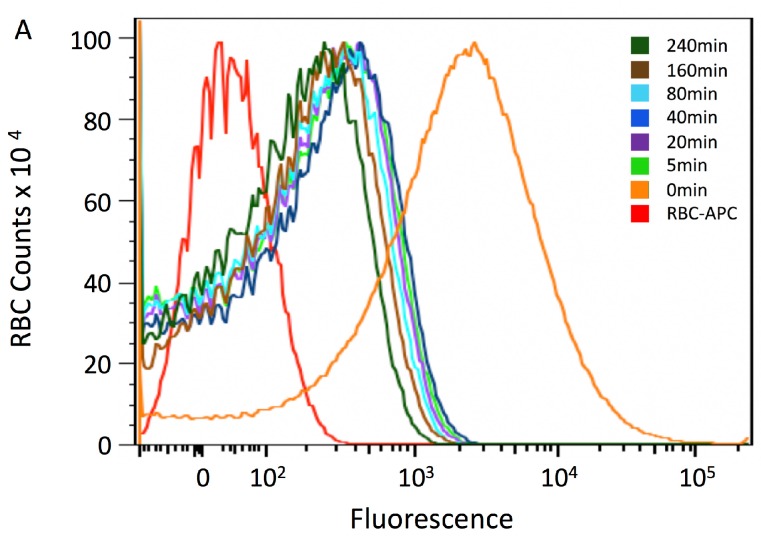

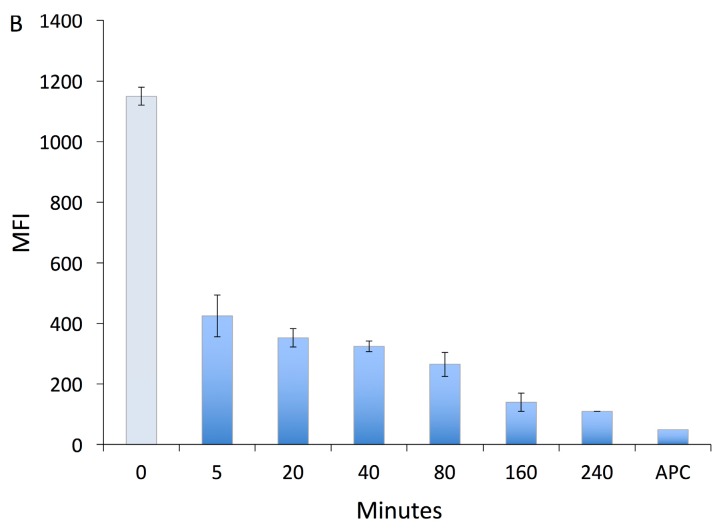
Time course of in vivo RBC adherence of BoNT/A complexed with 4-mAb:FP. The 4-mAb:FP complex bound to 200 ng BoNT/A (40,000 LD50) was injected i.v. into groups of five Swiss Webster mice, and their RBCs were tested for complex binding with an the APC anti-human IgG and flow cytometry. Blood samples were tested at 5, 20, 40, 80, 160 and 240 min, and the data for each group were averaged. For the zero timepoint, complexes were incubated with RBCs in vitro. (**A**) Flow cytometry tracings. (**B**) Calculated Mean Fluorescence Intensity (MFI) of the curves shown in Figure 4a. The zero timepoint value is depicted in light coloring to emphasize that it is not an in vivo sample. Error bars in Figure 4b indicate the S.D.

In our previous study, we found that conversion of the 6A and 4LCA mAbs into heteropolymers that adhered BoNT/A to the RBC surface enhanced their neutralization potency. However, the HP-containing BoNT/A complexes remained in the circulation for at least 2 h [8]. This is in contrast to polyclonal antibodies, which can clear toxin from the blood circulation within minutes [5,14]. We assessed the time course of BoNT/A clearance from the circulation induced by binding to the 4-mAb:FP complex (Figure 4). We bound 200 ng BoNT/A to the 4-mAb:FP in vitro, injected the complex intravenously into Swiss Webster mice, and obtained blood samples at the following timepoints: 5, 20, 40, 80, 160 and 240 min. Five mice were tested in each group. We assessed RBC binding of the complex to the RBCs using an APC anti-human IgG and flow cytometry. To measure an estimated zero timepoint value, assuming even distribution of the labeled complexes in vivo, prior to clearance, we also mixed the same BoNT/A complex with RBCs from 1.5 mL blood (one mouse equivalent) in vitro.

**Figure 5:**
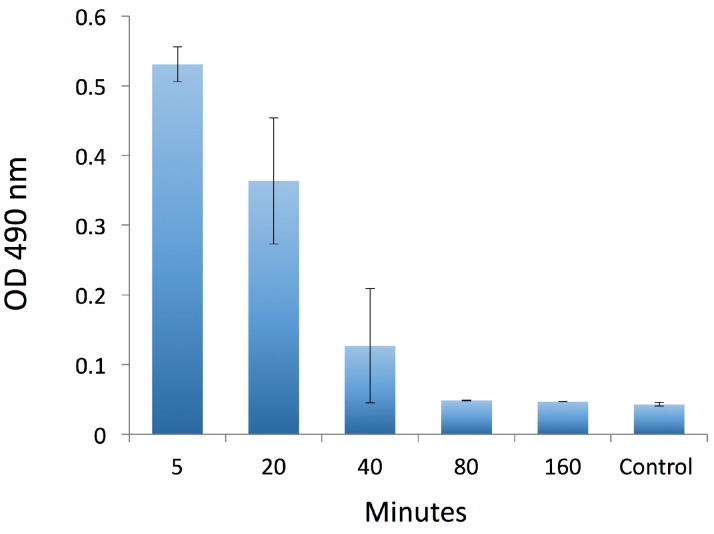
Detection of BoNT/A in plasma over time. In an experiment similar to the one shown in Figure 4, the 4-mAb:FP complex, bound to 200 ng BoNT/A (40,000 LD50), was injected i.v. into Swiss Webster mice. RI-BoNT/A in plasma was tested by ELISA at 5, 20, 40, 80, and 160 min in groups with 5 mice each. Control: dilution buffer only. Error bars indicate the S.E.M.

By the first in vivo timepoint tested, the mean fluorescence intensity (MFI) was 425 (Figure 4a,b), compared to 1150 at the estimated zero timepoint sample (in Figure 4b, the zero timepoint is shown in light blue). Labeling declined slowly thereafter, such that binding was still evident 4 h after injection of the complex, with an MFI of 110 (approximately 10% of the estimated labeling at time zero). This suggests that the complexes were cleared in a bi-phasic process. It is also notable that shapes of the curves did not change significantly between 5 and 240 min. We analyzed free BoNT/A in the plasma of these mice (Figure 5). Clearance of free BoNT/A followed a different time course, declining by less than half between the 5 and 20 min timepoints, suggesting that they represented a subset of the immune complexes that were defective in RBC binding and therefore cleared by a different mechanism.

### 2.3. Macrophage Uptake of mAb:FP Complexes In Vitro

**Figure 6:**
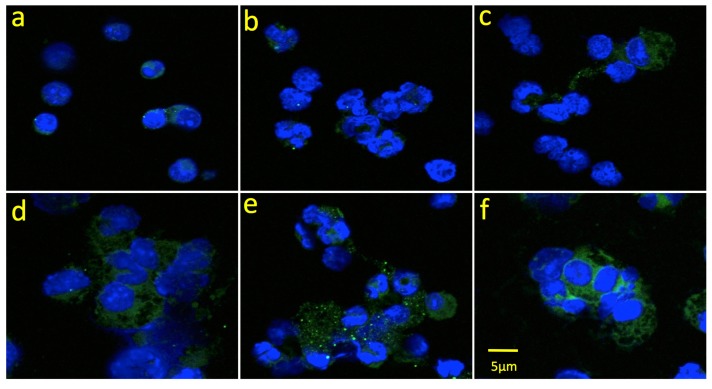
Phagocytosis of BoNT/A immune complexes by peritoneal macrophages. Alexa Fluor 488-labeled RI-BoNT/A was bound to the 3-mAb or 4-mAb combinations, alone or in combination with the FP or with FP and RBCs. Panel (**a**) RI-BoNT/A alone. Panels (**b**–**f**) RI-BoNT/A with: (**b**) 3-mAb, (**c**) 3-mAb + FP, (**d**) 4-mAb, (**e**) 4-mAb + FP, (**f**) 4-mAb + FP + RBCs.

**Figure 7:**
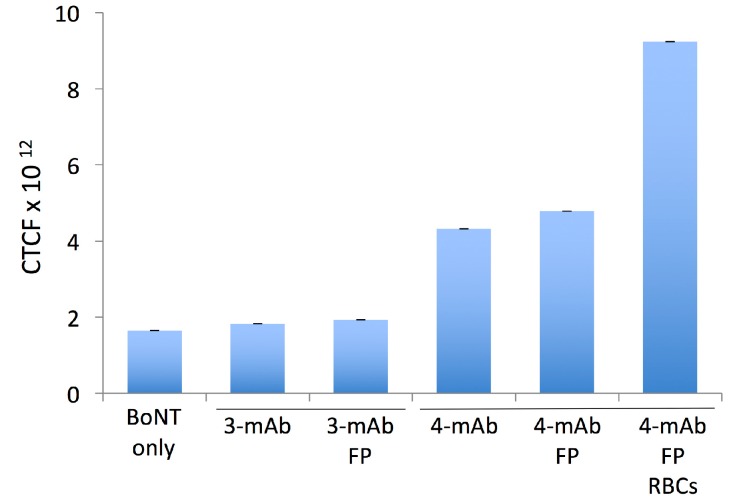
Quantitation of the uptake of RI-BoNT/A by macrophages in the experiment shown in Figure 6. Internalized Alexa-Fluor labeled RI-BoNT/A was assessed by measuring individual cells using confocal microscopy and IMAGEj software. The mean corrected total cell intensity (CTCF) is shown. Error bars indicate the S.E.M.

BoNT immune complexes in the bloodstream are cleared primarily by the liver, where they are taken up by Kupffer cells through Fc receptor-dependent mechanisms [3,15]. Kupffer cells share essential features with peritoneal macrophages, including expression of Fc receptors and Toll-like receptors, the ability to internalize immune complexes and present antigens, and the ability to polarize into diverse activation states [16]. Our previous study of HPs interacting with peritoneal macrophages showed that increasing the number of Fc domains (4 vs. 3 or 2) improved RI-BoNT/A uptake, and this observation correlated with neutralization potency in vivo [8]. Here, we tested the 3-mAb and 4-mAb combinations, with and without FP, for their ability to facilitate macrophage uptake of an Alexa 488-labeled RI-BoNT/A. We also tested the 4-mAb:FP in combination with RBCs. We assembled the complexes in vitro and incubated them with murine peritoneal macrophages for 30 min, then fixed and mounted the cells and analyzed them by confocal microscopy (Figure 6). In addition, we measured mean Corrected Total Cell Fluorescence (CTCF) using the Zeiss AIM 4.2 SP1 software with IMAGEj (Figure 7). RI-BoNT/A, alone or bound to the 3-mAb or 3-mAb:FP complexes, gave approximately equivalent uptake (Figure 6a–c and Figure 7). Greater uptake was seen with the 4-mAb and 4-mAb:FP complexes, although the FP-containing complexes had a less homogeneous cytoplasmic staining, with multiple, bright punctate signals in many of the cells (Figure 6d,e and Figure 7). The greatest uptake was seen with the 4-mAb:FP complexes bound to RBCs, which gave bright, homogeneous staining that was almost 2-fold over the level observed with the 4-mAb and 4-mAb:FP complexes (Figure 6f and Figure 7).

**Figure 8:**
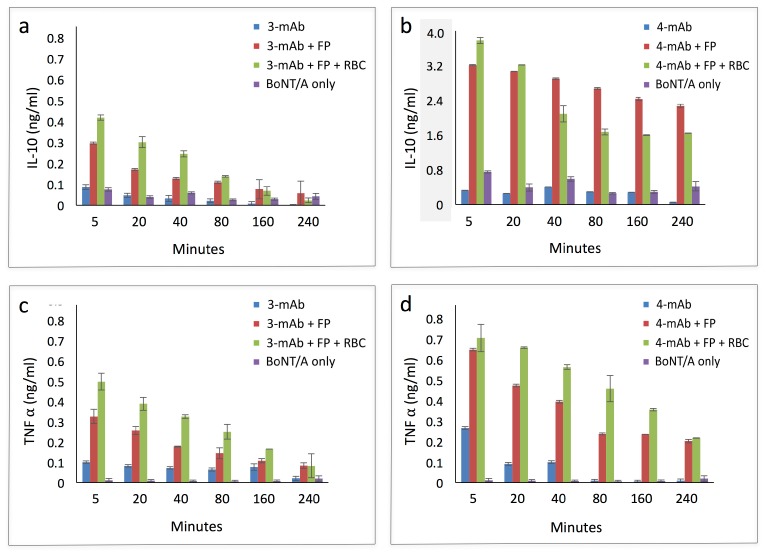
Cytokine production by peritoneal macrophages in the presence of RI-BoNT/A immune complexes and RBCs. Peritoneal macrophages were cultured in vitro and incubated with RI-BoNT/A bound to the 3-mAb or 4-mAb combinations, alone or in combination with the FP or with the FP and RBCs. The production of IL-10 and TNF-α was measured over time using a luminescence capture assay. Top panels: IL-10 production induced by complexes containing the (**a**) 3-mAb or (**b**) 4-mAb. Bottom panels: TNF-α production induced by complexes containing the (**c**) 3-mAb or (**d**) 4-mAb. The *y*-axis in panel (**b**) is greyed to emphasize that it has a different scale than panels (**a**,**c**,**d**).

Activated macrophages polarize into one of two activation phenotypes, classical (M1), characterized by expression of pathogen-killing nitric oxide and inflammatory cytokines such as IL-12 and TNF-α; and alternative (M2), an anti-inflammatory program associated with IL-10 expression [17,18]. Thioglycollate-mobilized murine peritoneal macrophages can be polarized in either direction [19], so we used them to test the effect of the 3-mAb:FP and 4-mAb:FP immune complexes on macrophage activation and polarization. We incubated 3-mAb and 4-mAb complexes bound to RI-BoNT/A (with and without RBCs) with thioglycollate-mobilized peritoneal macrophages. We tested the culture supernatants for expression of TNF-α and IL-10 (Figure 8, note the different *y*-axis scale in Figure 8b). RI-BoNT/A, alone or in complex with 3-mAb or 4-mAb, induced minimal TNF-α and IL-10 expression. In contrast, the FP substantially induced expression of both cytokines, whether in the presence of the 3-mAb or 4-mAb complexes, and whether RBCs were present or not. Furthermore, when combined with the 4-mAb, the FP induced IL-10 expression to a greater extent than TNF-α (induction of IL-10 ~9-fold vs. TNF-α ~1.4 fold at 5 min). Thus, the approximately 2-fold greater in uptake of the 4-mAb:FP complexes, relative to the 3-mAb:FP complexes, was accompanied by a disproportionately elevated IL-10 induction. Furthermore, the doubling of uptake provided by RBC binding of the 4-mAb + FP complex did not correlate with a proportionate increase in IL-10 expression.

## 3. Discussion

Studies of the protective efficacy of mAbs against diverse toxins have established the general principle that combinations of mAbs generally provide better in vivo protection than individual mAbs [20,21]. Oligoclonal mAbs can provide some of the benefits of the authentic polyclonal anti-toxin antibody response, including enhanced inhibition of toxin activity, improved binding affinity, alteration of toxin conformation, and improved clearance in vivo [22,23,24,25]. However, the creation of protective mAb combinations for use as therapeutics is challenged by the observation that the majority of anti-toxin mAbs described have no intrinsic neutralizing activity [26], and that mAb combinations do not predictably improve neutralization in vivo relative to single mAbs [23,24]. Therefore, methods to enhance the sequestration and/or clearance of toxins in vivo may have general utility in improving the efficacy of oligoclonal mAb anti-toxins. 

The study of BoNT neutralization by mAbs offers a general paradigm for neutralization of toxins, especially those that pass through the blood circulation during the intoxication process, such as Shiga toxins, staphylococcal enterotoxins, ricin, and anthrax toxin [27,28,29,30]. To use mAbs as a countermeasure for BoNT intoxication, we need to address three fundamental issues. First, BoNT exists in at least seven different serotypes [31]. Second, three or more mAbs need to bind BoNT simultaneously in order to obtain therapeutic neutralization efficacy in vivo [4,5]. Third, mAbs that bind to multiple serotypes generally do not have substantial neutralization potency [32]. These factors complicate efforts to create a comprehensive panel of anti-BoNT mAbs, so mAb-based BoNT countermeasures need to inhibit as many parts of the intoxication process as possible [2]. Here, we have continued our studies of how RBC adherence may improve the in vivo BoNT neutralizing capacity of mAb combinations.

We identified a combination of 4 mAbs that work with the FP to give a high level of in vitro neutralization. We then studied subsets of that combination to identify factors that contribute to BoNT neutralization in vivo. When administered without the FP, the neutralization potency increased with the number of mAbs in the complex (2-mAb vs. 3-mAb vs. 4-mAb). This result correlated with the finding that macrophages took up the 4-mAb complex better than the 3-mAb complex. The addition of the FP increased the neutralization potency of each combination, but it had its greatest effect when 4 mAbs were present. In the macrophage uptake experiments, the FP by itself did not increase complex uptake. However, in the presence of RBCs, macrophage uptake of the 4-mAb complex was doubled. This phenomenon reflects the known ability of RBCs to accelerate the transfer of complement-bound immune complexes to macrophages and may explain some of improvement provided by the FP in vivo [33]. 

The rapid clearance of the immune complexes from the RBC surface in vivo correlated with previous observations with polyclonal antisera, and was consistent with the speed with which macrophages can engage and phagocytose immune complexes presented by RBCs [3,34]. More than half of the injected BoNT/A was cleared from the blood circulation by the time the first in vivo timepoint could be assessed, and complexes may have been cleared by RBC-adherent or RBC-independent processes. It is unlikely that the initial drop in BoNT/A in the circulation related to tissue distribution, as has been observed with BoNT/A administered alone, because this process has a half-life of ~60 min. It is also possible that some of the BoNT/A immune complexes were cleared without RBC binding, but the substantial improvement in neutralization provided by the FP suggests that RBC binding did occur in vivo.

In our previous studies of mAbs in the heterodimeric HP format, we found that two HPs, which contributed a total of 4 Fc domains to the complex, were taken up more rapidly by macrophages than an HP and a single mAb (3 Fc domains), and that this correlated with neutralization potency in vivo [8]. These findings and the present study are consistent with a model for Fc-receptor dependent IgG uptake in which a threshold level of receptor engagement is required to induce a phagocytic response of the FP-bound complexes [35,36]. This all-or-nothing response is directly related to IgG density-dependent activation of intracellular signaling pathways, enhanced by a beneficial display of the immune complexes on the RBC surface [33]. Previous studies of BoNT-neutralizing mAbs have shown that 3 mAbs can be sufficient for high level neutralization in vivo [4], but all of the mAbs studied in Nowakowski et al. bound to the BoNT/A HC domain, which may have placed them in an optimum geometry for macrophage uptake. It is possible that the orientation of Fc domains in an immune complex may affect the ease with which they collaboratively induce Fc receptor cross-linking on the macrophage surface.

The shape of the flow cytometry curves in this experiment revealed a broad heterogeneity of labeling that included a substantial proportion of completely unlabeled RBCs. This may have resulted from incomplete mixing during injection. Alternatively, these shapes would also be consistent with a model in which multiple BoNT immune complexes are removed from the RBCs simultaneously during the short period of time in which the density of the immune complexes on the RBC surface are sufficient to trigger phagocytosis. The level of complex clearance would therefore be a stochastic process determined by extent of macrophage engagement of the RBC membrane during the phagocytic process [37].

After the initial rapid phase of BoNT/A immune complex clearance, the remaining complexes appeared to stabilize on the RBC surfaces, as they remained bound for many hours and the flow cytometry tracings did not re-distribute into bell-curve shapes. This is consistent with the observation that BoNT/A in the plasma was below the limit of detection by 80 min, i.e., essentially entirely adherent to the RBCs. In addition, their existence on the RBC surface for so long, in comparison to capability of macrophages to rapidly take up complexes in vitro and remove them from the plasma in vivo [34] suggests that the complexes in the second clearance phase were in a configuration that could not be taken up by macrophages. Our provisional model is that the concentration and/or RBC binding density are sufficient for efficient macrophage uptake in the first phase of clearance, but not in the second.

Although the FP by itself did not improve the uptake of the 3-mAb and 4-mAb complexes by the peritoneal macrophages, it did contribute to macrophage activation, as measured by induced TNF-α and IL-10 expression. Notably, the FP and the 4-mAb complex had a strong synergistic effect on IL-10 expression. No comparable synergy was seen with TNF-α expression, indicating that the addition of the FP to the 4-mAb:BoNT/A complex induced M2 polarization of the peritoneal macrophages. Macrophages can take up immune complexes without activation, but they may undergo M2 polarization in the presence of high density IgG complexes and Toll-like receptor (TLR) activation. Notably, M2 polarization is typical of the liver macrophages that are likely to be determinants of BoNT clearance in vivo. Liver macrophages are subject to continuous TLR4 activation from endotoxin, which combines with immune complex stimulation to promote a non-inflammatory, tolerogenic phenotype [38]. In the present study, it is possible that the streptavidin moiety in the FP may have provided a TLR-activating effect to trigger macrophage polarization in the presence of the larger immune complex, as it did not increase the levels of endotoxin present in the macrophage supernatants (data not shown).

The improvement in BoNT/A neutralization provided by the FP likely results from the combined effects of sequestration of BoNT/A on the RBC surface as well as improved complex clearance in the immediate post-exposure period. Although the effect of the FP on macrophage activation was substantial, it did not correlate with improved uptake by macrophages. The benefits of immediate toxin sequestration and improved toxin clearance provided by the FP are countered by the fact that some BoNT/A remained on the RBC surface for many hours. In the present series of experiments, this was balanced by the use of mAbs with high intrinsic BoNT neutralizing activity. 

Because mice do not express an RBC complement receptor protein, instead using a platelet-expressed protein for immune adherence, we have been able to somewhat isolate the effects of FP-mediated RBC binding on BoNT neutralization. Nonetheless, a caveat of the present study is that non-RBC-mediated clearance from the blood circulation may have proceeded in parallel to FP-mediated clearance. Additional studies will need to assess how enhanced RBC adherence would be best optimized in for humans, in whom macrophage uptake is a collaboration between Fc receptor interactions and adhesive contributions provided by the complement receptor [39].

## 4. Conclusions

- Botulinum toxin neutralization in vivo by IgG immune complexes can be improved by fusion protein-mediated adherence to red blood cells (RBCs).
- RBC adherence, mediated by complex binding to a fusion protein (FP), which is a fusion between streptavidin and an anti-glycophorin A scFv, sequesters botulinum neurotoxin from the neuromuscular junction.
- Botulinum toxin-containing immune complexes that have adhered to RBCs through the FP show increased uptake by macrophages and induce M2 macrophage polarization.
- Clearance of immune complexes bound to RBCs through an FP shows bi-phasic kinetics, with most clearance occurring within the first five minutes, and the rest declining slowly thereafter.

## 5. Materials and Methods

### 5.1. Botulinum Neurotoxin, Fusion Protein, Monoclonal Antibodies and Immune Complex Assembly

Pure Type A1 botulinum neurotoxin (BoNT/A) was obtained from Metabiologics (Madison, WI, USA). We performed all of the experiments with a single lot of toxin, which we assayed for its neurotoxin content using the mouse protection assay, and measured the single LD50 to be 5 pg [12]. The BoNT/A stock was distributed into working aliquots and stored at −20 °C. Purified rabbit anti-BoNT/A polyclonal IgG and recombinant inactive BoNT/A (RI-BoNT/A) were gifts from Inventox, Inc. (Santa Fe, NM, USA) [40,41]. The 6A and 3B3 human mAbs, which bind the BoNT/A heavy chain (HC), and the 4LCA mAb, which binds the BoNT/A light chain (LC), were previously described [9,42]. The CR2 mAb, specific for the N-terminal domain of the BoNT/A heavy chain [10], was obtained from Dr. Jianlong Lou and Dr. James Marks (University of California, San Francisco, CA, USA). The Fusion Protein (FP) was produced in *E. coli* expression as described previously [9].

### 5.2. Animals and BoNT/A Neutralization Testing 

Female 6–8 week-old Swiss Webster mice were purchased form Taconic Biosciences (Germantown, NY, USA) and housed at the AAALAC-certified animal facility at the Lankenau Institute for Medical Research. All the mice had free access to food and water. All procedures were approved by Lankenau Institute for Medical Research Animal Care (Protocol No: A08-2692, Approval Period: 28 July 2016–27 July 2017) and Use Committee IACUC. For in vivo testing of BoNT/A neutralization, mice were sedated with isoflurane prior to intravenous injection with the mAb and mAb:FP combinations. Mice were monitored hourly for 6 h post-injection, and then twice daily for up to seven days. Mice exhibiting signs of BoNT/A intoxication, such as paralysis, cachexia, hunched backs, eye secretions, rapid breathing, or hypokinesis were euthanized by CO_2_ inhalation.

### 5.3. Monoclonal Antibody Competitive Binding Studies

Black Nunc Maxisorp plates (Nalgene Nunc International, Rochester, NY, USA) were coated overnight with 5 μg/mL 4LCA (Figure 1A–D) or rabbit anti-BoNT/A HC50 antiserum in phosphate buffer saline (PBS) followed by three washes in PBS containing 0.05% Tween 20 (PBST). The plates were blocked with 2% non-fat milk in PBST for 1 h at 37 °C, followed by three PBST washes, and then serial dilutions of RI-BoNT/A in plasma (50 μL each), incubated for 1 h at 37 °C, followed by a 3× PBST wash. Either PBS or 5 μg/mL of the biotinylated mAb (6A or 3B3), diluted in PBS, were added to the samples, incubated for 1 h at 37 °C, and washed 3× in PBST. Streptavidin-poly-horseradish peroxidase conjugate (Thermo Fisher Scientific, Waltham, MA, USA) was added (1:2000), incubated for 1 h at 37 °C, and washed 3× with PBST. Super Signal ELISA Femto Substrate (Thermo Fisher Scientific, Waltham, MA, USA) was used for detection and relative luminescence values were measured using Biotek Synergy II Microplate reader (BioTek Instruments, Winooski, VT, USA). Excel was used to process the data. MAbs were biotinylated using the EZ-Link™ Hydrazide-Biotin kit (Thermo Fisher Scientific, Waltham, MA, USA) according to the manufacturer’s instructions. Protein concentration was measured using the NanoDrop 1000 (Thermo Fisher Scientific, Waltham, MA, USA).

### 5.4. Immune Complex Assembly and RBC Binding In Vitro and In Vivo

To assemble the FP + mAb containing immune complexes, we used a sequential binding protocol. We incubated the specified dose of BoNT/A or RI-BoNT/A with 20 μg 4LCA and 20 μg 6A for 1 h at room temperature. This was followed by 10 μg 3B3 and 2 μg CR2 mAbs (if needed), another hour of incubation, then 30 μg FP (if indicated), followed by another 30 min incubation. For the in vitro RBC binding experiments, Swiss Webster mouse blood (1.5 mL) was collected in heparinized tubes and centrifuged for 20 min at 8000 RPM, and the RBC pellet was washed 3× in 1000 μL PBS:1% BSA. The pellet was resuspended in 3600 μL PBS-BSA, divided into three aliquots, and rocked gently at room temperature until use. RBCs were gently rocked with the indicated complexes for 1 h, centrifuged for 8 min at 5000 RPM, washed 3 times with 1% BSA-PBS, and incubated with APC-conjugated F(ab’)2 donkey anti-human IgG (Jackson ImmunoResearch, West Grove, PA, USA) for 30 min at room temperature. Cells were washed twice in 1% BSA-PBS and quantified with a BD FACSCanto II (Becton Dickson, Franklin Lakes, NJ, USA). Data were analyzed using FlowJo 8.8.6. Software (Tree Star, Ashland, OR, USA). The presence of BoNT/A in the supernatant before and after RBC binding was assessed by ELISA with the rabbit anti-BoNT/A HC50 antiserum with OPD as the chromogenic substrate, and absorbance was determined at 490 nm [13,43]. 

For the in vivo RBC binding and clearance experiments, 4-mAb:FP bound in vitro to 200 ng RI-BoNT/A was injected i.v. into Swiss Webster mice (Taconic Biosciences, Germantown, NY, USA) under isoflurane anesthesia. Mice were bled retro-orbitally at 5, 20, 40, 80, 160, and 240 min post injection. A “time zero” timepoint was obtained by mixing the complex with one mouse-equivalent of RBCs in vitro. Blood was spun for 5 min at 3000 RPM, then the RBCs were washed and analyzed by flow cytometry using the anti-human IgG APC secondary antibody. RI-BoNT/A in the plasma was assayed by ELISA as described above. Five mice were tested in each group and each experiment was performed twice. 

### 5.5. Macrophage Interaction with BoNT/A Immune Complexes 

Swiss Webster mice were received intraperitoneal injections of sterile 3% Thioglycollate Medium, Brewer Modified solution (Thermo Fisher Scientific, Waltham, MA, USA) under 3% isoflurane anesthesia for 3–5 min. After 4 days, peritoneal macrophages were collected using cold PBS, centrifuged at 1000 rpm for 10 min at 4 °C, and washed with Dulbecco’s modified eagle medium (DMEM) containing 20% fetal bovine serum, 100 U/mL penicillin and 100 μg/mL streptomycin (Thermo Fisher Scientific, Waltham, MA, USA). 1 million cells were plated on cover slips in 24 well tissue culture plates and incubated at 37 °C. After 2 h, non-adherent cells were removed and the plate washed 3 times with warm DMEM. 

15 ng Alexa Flour 488 labeled RI-BoNT/A DyLight microscale labeling kit (Thermo Fisher Scientific, Waltham, MA, USA) were assembled in mAb or mAb:FP immune complexes as described above, and bound to 100 μL washed RBCs in vitro for 30 min where indicated. The RI-BoNT/A-containing immune complexes were incubated with the macrophages at 4 °C for 30 min and then an additional 30 min at 37 °C. The cells were washed with serum free medium 3 times, fixed with 4% paraformaldehyde solution (Sigma-Aldrich, St. Louis, MO, USA) for 30 min at 4 °C, and washed 3 times with PBS. The cover slips were mounted on microscope slides with Prolong Gold antifade reagent with 4′,6-diamidino-2-phenylindole (DAPI) (Thermo Fisher Scientific, Waltham, MA, USA). Images were acquired using a Carl Zeiss LSM 510 UV META inverted confocal microscope with a Plan-Apo 40× oil immersion lens (Zeiss Microimaging, Thornwood, NY, USA), at room temperature and Zeiss AIM 4.2 SP1 software (Zeiss Microimaging, Thornwood, NY, USA).

## Figures and Tables

**Table 1:** Neutralization of BoNT/A in vivo by mAb and mAb:FP complexes. The mAbs and mAb:FP combinations were tested for neutralization in mice. Complexes were formed in vitro prior to intravenous injection into groups of Swiss Webster mice. The numbers of mice surviving/numbers of mice tested are given for each dose of BoNT/A.

Complex	5000 LD50	7500 LD50	10,000 LD50	20,000 LD50	40,000 LD50	Components
2-mAb	0/10	0/10				6A + 4LCA
2-mAb:FP	10/10	0/10	0/10			6A + 4LCA + FP
3-mAb	2/10	0/10	0/10			6A + 4LCA + 3B3
3-mAb:FP	20/20	6/20	0/10	0/5		6A + 4LCA + 3B3 + FP
4-mAb	20/20	6/20	0/10	0/5		6A + 4LCA + 3B3 + CR2
4-mAb:FP	10/10	10/10	10/10	20/20	20/20	6A + 4LCA + 3B3 + CR2 + FP

## Abbreviations

2-mAb	4LCA + 6A mAbs
3-mAb	4LCA + 6A + 3B3 mAbs
4-mAb	4LCA + 6A + 3B3 + CR2 mAbs
BoNT/A	botulinum neurotoxin type A
CR1	complement receptor 1
CTCF	corrected total cell fluorescence
FP	fusion protein
GPA	glycophorin A
HC	BoNT heavy chain
HC50	a recombinant 50 KD amino-terminal domain of BoNT/A
HP	heteropolymer
IL-10	interleukin-10
LC	BoNT light chain
LD50	mouse 50% lethal dose
M1	classical macrophage polarization
M2	alternative macrophage polarization
mAb	monoclonal antibody
MFI	mean fluorescent intensity
NMJ	neuromuscular junction
RI-BoNT/A	recombinant inactive BoNT/A
RBC	red blood cells
S.D.	Standard Deviation
S.E.M.	Standard Error of the Mean
TLR	toll-like receptor
TNF-α	tumor necrosis factor-α
